# Association between selective IgA deficiency and COVID-19

**DOI:** 10.3164/jcbn.20-102

**Published:** 2020-08-21

**Authors:** Yuji Naito, Tomohisa Takagi, Tetsuro Yamamoto, Shaw Watanabe

**Affiliations:** 1 Department of Gastroenterology and Hepatology, Kyoto Prefectural University of Medicine, 465 Kajii-cho, Kamigyo-ku, Kyoto 602-8066, Japan; 2 TTC Co. Ltd., 1-20-2 Ebisu-nishi, Sibuya-ku, Tokyo 150-0021, Japan; 3 Asia Pacific Clinical Nutrition Society, Life Science Promoting Association, 25-3-1004 Daijyo-cho, Shinjuku, Tokyo 160-0015, Japan

**Keywords:** COVID-19, IgA deficiency, mortality

## Abstract

The purpose of this study was to propose a hypothesis that there is a potential association between the incidence of selective IgA deficiency in various countries and COVID-19 cases. The number of deaths due to COVID-19 increased in clear proportion to the number of infected patients, and the difference in the number of deaths by country was due to the difference in the number of infected patients. The frequency of selective IgA deficiency has a strong positive correlation with the prevalence of COVID-19 per population. The low infection rate contributed to the low death rate from COVID-19 in Japan, suggesting that the extremely low frequency of selective IgA deficiency may be a contributing factor.

## Introduction

The new infectious coronavirus disease (COVID-19) has spread worldwide; however, it has been reported that the number of deaths from COVID-19 is relatively small in the East Asian region, including Japan, compared to the Western countries.^(1)^ According to the website information on June 12, 2020, the number of deaths varies widely by region and country. Among them, it has been noted that the number of COVID-19 deaths in Japan is extremely small, and some hypotheses have been proposed to explain this occurrence. However, so far, these explanations are not clear. In order to propose long-term actions against COVID-19, it is crucial to organize the information obtained at present and assess the factors related to COVID-19.

The IgA, which is the most produced antibody in the body, functions at the forefront of the biological defense mechanism in infections that target mucosal tissues such as influenza.^(2)^ Secretory IgA is an antibody that is produced in large amounts in mucus located on the surface of mucosal epithelium, and makes first contact with antigens in the mucosal defense mechanism. By binding IgA to bacteria, viruses, and toxins, adhesion of bacteria and viruses to epithelial cells as well as uptake of toxins is blocked, thus, providing the first line of defense against various pathogens. However, so far, there are no reports on the association between COVID-19 infection and IgA. The purpose of this study was to compare information on COVID-19 by country, and to clarify its association with selective IgA deficiency.

## Methods

COVID-19 information on the number of infected people and deaths by country was obtained from the “COVID-19 Dashboard” on the Center for Systems Science and Engineering (CSSE) website,^(3)^ and the population of each country was obtained from the Global Note website^(4)^ on June 12, 2020. The national frequency of selective IgA deficiency was obtained on the same day from PubMed,^(5)^ using “selective IgA deficiency” as a keyword.

## Results

Table 1 presents the number of COVID-19 infections and deaths, populations, and frequencies of selective IgA deficiency in 19 countries.

### Correlation between the number of patients infected with COVID-19 and the number of deaths

**Fig. 1:**
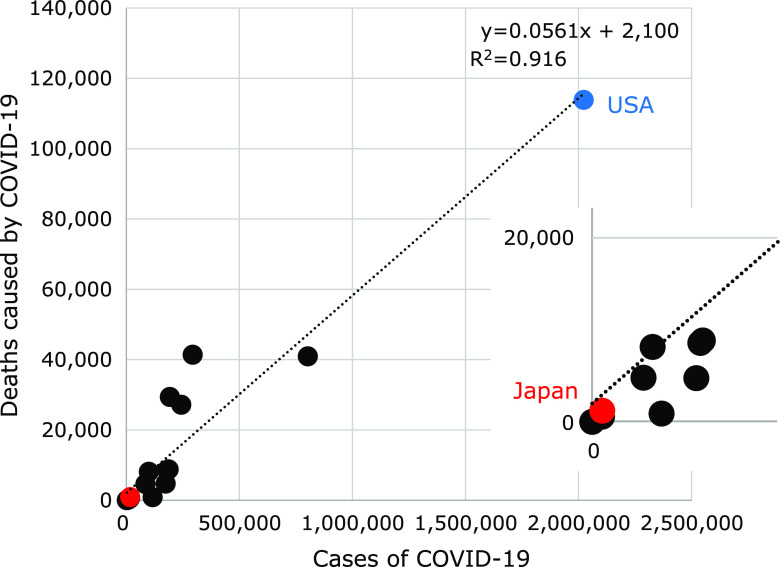
Correlation between the number of patients infected with COVID-19 and the number of deaths.

As shown in Fig. 1, the number of deaths increased in proportion to the increase in the number of people infected with COVID-19, and there was a clear positive correlation between the two. The correlation coefficient showed that 5% of those who were infected died from the disease.

### Correlation between the number of patients infected with COVID-19 and the mortality rate

**Fig. 2:**
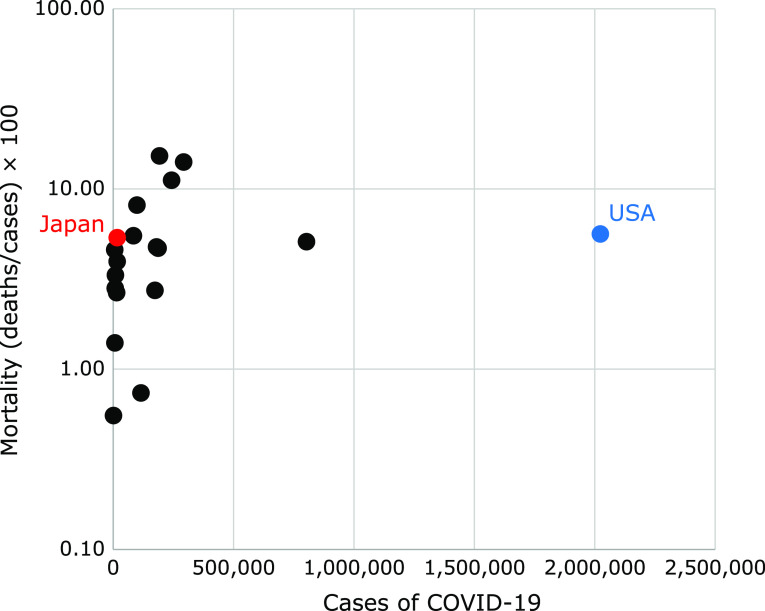
Correlation between the number of patients infected with COVID-19 and the mortality rate.

As shown in Fig. 2, there was no correlation between COVID-19 infections and mortality. The number of infections in the USA is the highest at 2,023,385, and in Japan, the number is as low as 17,187, which is 0.85% that of the USA. However, the mortality rate is 5.63% in the USA and 5.36% in Japan, indicating no significant difference.

### Correlation between the COVID-19 infection rate and frequency of selective IgA deficiency

As firstly pointed out,^(6)^ the relative proportion of individuals affected by secretary IgA deficiency varies among countries. Table 1 showed the incidence of selective IgA deficiency calculated from representative data in each country, which were obtained from the literatures.^(7–10)^ The incidence varies from 1:143 to 1:14,800. In general, IgA deficiency is more common in Caucasians. In the USA, the frequency is estimated to be from 1:333 to 1:3,000 among healthy blood donors.^(11)^ In this study, the frequency in the USA was used as 0.300%. Of the blood donors screened in Japan, only 0.007% (1:14,840) were found to be IgA-deficient (less than 10 mg/dl) by means of the double diffusion method.^(12)^

**Fig. 3:**
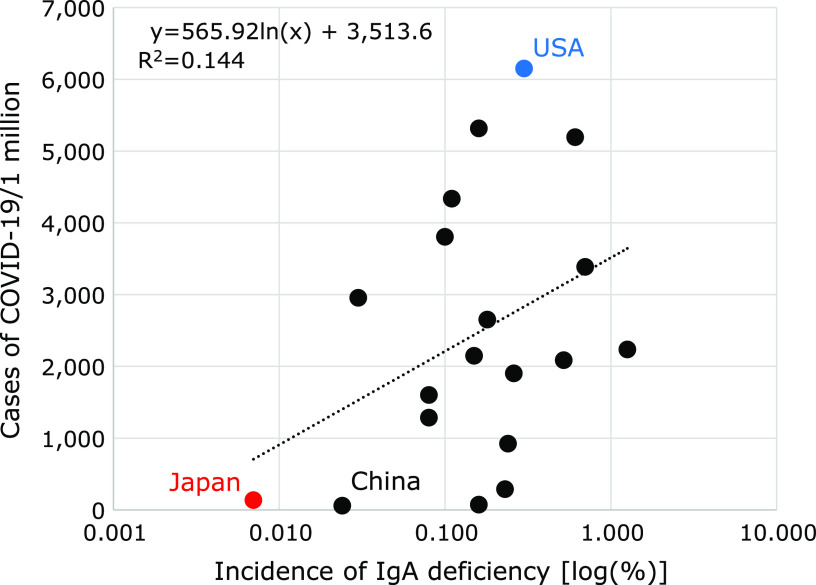
Correlation between the COVID-19 infection rate and frequency of selective IgA deficiency.

As shown in Fig. 3, there was a positive correlation between the frequency of selective IgA deficiency and the COVID-19 infection rate per 1 million population. It should be noted that the low COVID-19 infection rate in China is under-calculated due to the different definition of COVID-19 infection applied in China.

## Discussion

This research is the result of analyzing the information obtained on June 12, 2020. The most important result of this study is that the number of deaths due to COVID-19 increased in clear proportion to the number of infected patients, and the difference in the number of deaths by country was due to the difference in the number of infected patients. COVID-19 has been identified as an infectious disease that kills 1 in 20 people. In other words, the reason for the low number of deaths from COVID-19 in Japan is probably due to the low infection rate per population. Although the low number of polymerase chain reaction (PCR) tests for SARS-CoV-2 has been a problem in Japan and overseas, it is unlikely that there are many undiagnosed infected people. The results of a recent SARS-CoV-2 antibody screening test in a general population also show the distinctly different COVID-19 infection rates between the USA and Japan. Elucidating the causes of COVID-19 infection rates in different countries is important for proposing long-term actions against this infection.

Another important finding in this study was a strong positive correlation between the frequency of selective IgA deficiency and the prevalence of COVID-19 infection per population. The frequency of selective IgA deficiency in the USA was more than 40 times that of Japan. The high frequency of selective IgA deficiency in the Western countries suggests that the heterogeneous genotype population is even higher, and that the number of COVID-19 infections increases proportionately, resulting in an increase in deaths.

Recent studies have revealed the importance of secretory IgA in mucosal defense, and contain important information for understanding the pathophysiology of COVID-19 infection. First, it was reported that the production of secretory IgA in respiratory tract mucosa and intestinal mucosa was impaired in patients with COVID-19.^(13)^ Second, drug-induced secretory IgA deficiency has been identified. So far, cases in which drugs such as sulfasalazine, d-penicillamine, gold, phenytoin, valproic acid, thyroxine, captopril, levamisole, and cyclosporine cause secretory IgA deficiency have been identified. In addition, infectious diseases such as cytomegalovirus, rubella, toxoplasmosis, and Epstein Barr virus are also considered to cause secretory IgA deficiency.^(13)^ Recently, we reported a severe case of COVID-19 infection complicated with cytomegalovirus infection.^(14)^ Third, epidemiologic studies of selective IgA deficiency have revealed that recurrent respiratory infections are the most common.^(10)^ To the best of our knowledge, so far, no detailed information is available on secretory IgA with regards to COVID-19.

Furthermore, the results of this study suggest that the strategy of infection prevention by promoting the production of secretory IgA may be important. For example, probiotics that enhance the production of secretory IgA antibodies have also been identified. A double-blind comparative study was conducted in which *Lactobacillus delbrueckii* ssp. *Bulgaricus *R-1 strain was administered for 12 weeks, and these probiotics significantly increased IgA production in saliva in response to the influenza A H3N2 virus.^(15)^ Strengthening the mucosal barrier with probiotics and prebiotics may increase resistance to viral infections in the host, and future research with this regard is expected.

Vaccine development is important for long-term management against COVID-19. Among various vaccines, immunization with a nasal vaccine has some advantages. A nasal vaccine is not only non-invasive but also capable of inducing IgA with virus neutralizing activity. Furthermore, a nasal vaccine technique with crossability against multiple strains of the influenza virus has been developed by efficiently producing not only dimeric IgA but also tetrameric IgA.^(16)^ A nasal vaccine may also be effective against the SARS-CoV-2 gene mutant strain, and has a feature that is not present in the IgG-inducing vaccine.

The present study has several limitations. First, information on the frequency of selective IgA deficiencies was only available in 19 countries. We would like to have access to information on East Asian countries including South Korea, where effective preventive measures against COVID-19 infections are implemented. Second, this study is based only on epidemiological information, and studies on the molecular mechanism of IgA in the prevention of COVID-19 are needed.

## Conclusion

The number of deaths from COVID-19 infections increased in clear proportion to the number of infected patients, and there is no significant difference in the mortality rate among countries. There was a strong positive correlation between the frequency of selective IgA deficiency and the COVID-19 infection rate per population. The low infection rate contributed to the low death rate from COVID-19 infection in Japan, suggesting that the extremely low frequency of selective IgA deficiency may be a contributing factor.

## Figures and Tables

**Table 1:** COVID-19 cases, its mortality, and the incidence of selective IgA deficiency in various countries

Countries	Population (M)	Cases of COVID-19	Deaths caused by COVID-19	Cases of COVID-19/1M	Deaths caused by COVID-19/1M	Mortality (deaths/cases) ×100	IgA deficiency (%)
Australia	25.20	7,288	102	289.2	4.0	1.3996	0.230
Austria	8.95	17,034	674	1,903.2	75.3	3.9568	0.260
Brazil	211.05	802,828	40,919	3,804.0	193.9	5.0969	0.100
Canada	37.41	99,159	8,071	2,650.6	215.7	8.1395	0.180
China	1,433.78	84,216	4,638	58.7	3.2	5.5073	0.024
Czechia	10.69	9,855	328	921.9	30.7	3.3283	0.240
Finland	5.50	7,064	325	1,284.4	59.1	4.6008	0.080
France	65.13	192,493	29,349	2,955.5	450.6	15.2468	0.030
Germany	83.52	186,691	8,772	2,235.3	105.0	4.6987	1.260
Icelans	0.34	1,807	10	5,314.7	29.4	0.5534	0.160
Iran	83.91	180,156	8,584	2,147.0	102.3	4.7648	0.150
Japan	126.86	17,187	922	135.5	7.3	5.3645	0.007
Nigeria	200.96	14,554	387	72.4	1.9	2.6591	0.160
Norway	5.38	8,608	242	1,600.0	45.0	2.8113	0.080
Sauji Arabia	34.27	116,021	857	3,385.5	25.0	0.7387	0.700
Spain	46.74	242,707	27,136	5,192.7	580.6	11.1806	0.610
Turkey	83.43	174,023	4,763	2,085.9	57.1	2.7370	0.520
UK	67.53	292,860	41,364	4,336.7	612.5	14.1242	0.110
USA	329.06	2,023,385	113,818	6,149.0	345.9	5.6251	0.300

M, million.
